# Intravitreal faricimab pharmacokinetics assessed by PET imaging in a neovascular Age-related Macular Degeneration rat model

**DOI:** 10.1016/j.ijpx.2026.100567

**Published:** 2026-05-14

**Authors:** Xurxo García-Otero, Andrea Cuartero-Martínez, Patricia Fernández-Robredo, Soraya Groba-de Antas, Noemí Gómez-Lado, Francisco J. Otero-Espinar, María Hernández, Jaione Bezunartea, Alfredo García-Layana, Sergio Recalde, Pablo Aguiar, Anxo Fernández-Ferreiro

**Affiliations:** a Molecular Imaging and Pharmacokinetic Modelling Group, Center for Research in Molecular Medicine and Chronic Diseases (CiMUS), University of Santiago de Compostela, Spain; b Molecular Imaging Group and Nuclear Medicine, Health Research Institute of Santiago de Compostela (IDIS), University Hospital Santiago de Compostela, Spain; c FarmaCHUSLab Group, Health Research Institute of Santiago de Compostela (IDIS), Santiago de Compostela, Spain; d Ophthalmology Experimental Laboratory, Navarra Institute for Health Research (IdiSNA), University of Navarra, Pamplona, Spain; e Department of Ophthalmology, Clínica Universidad de Navarra, Pamplona, Spain; f Nano-Oncology and Translational Therapeutics Group, Health Research Institute of Santiago de Compostela (IDIS), University Hospital Santiago de Compostela, Spain; g Department of Pharmacology, Pharmacy and Pharmaceutical Technology, Faculty of Pharmacy, University of Santiago de Compostela (USC), Santiago de Compostela, Spain; h Paraquasil Group, Health Research Institute of Santiago de Compostela (IDIS), University Hospital Santiago de Compostela, Spain; i Institute of Materials (iMATUS), University of Santiago de Compostela (USC), University Hospital Santiago de Compostela, Spain; j Hospital Pharmacy Department, University Hospital Santiago de Compostela, SERGAS, Spain

**Keywords:** Faricimab, Pharmacokinetics, Biodistribution, CNV model, Anti-VEGF, Anti-ANG-2

## Abstract

Age-related Macular Degeneration (AMD) is the leading cause of blindness in the elderly, with its neovascular form characterised by abnormal vessel growth. Current anti-VEGF therapies require frequent intravitreal injections and show variable efficacy. Faricimab, a novel bispecific antibody targeting VEGF-A and ANG-2, offers a dual mechanism with the potential for enhanced efficacy and extended dosing intervals. This study aimed to characterise the intravitreal pharmacokinetics, biodistribution, and ocular localization of faricimab in a rat model of laser-induced choroidal neovascularization (CNV) using non-invasive molecular imaging. Faricimab was conjugated with DFO and radiolabelled with zirconium-89 ([^89^Zr]Zr-DFO-faricimab), maintaining functional binding to VEGF-A and ANG-2. Following intravitreal injection, pharmacokinetics was assessed by PET/CT, blood sampling, and autoradiography, alongside structural retinal evaluation by OCT. Both healthy and CNV-induced rats demonstrated biphasic ocular clearance with similar half-lives, obtaining no significant differences (Control AUC_₀–∞_ = 5587.3 ± 1419.3%·hour and AMD AUC_₀–∞_ = 4079.1 ± 1178.6%·hour). Autoradiography confirmed predominant retention in the posterior segment, with AMD eyes showing higher early accumulation, suggesting altered diffusion, and increased retinal binding. Whole-body biodistribution revealed systemic uptake primarily in the liver and spleen, consistent with antibody catabolism. This work represents the first study to report pharmacokinetic data of faricimab as well as its intraocular distribution in an AMD model. Our findings support its therapeutic potential and highlight PET imaging as a powerful non-invasive tool for longitudinal ocular pharmacokinetic studies.

## Highlights

• First non-invasive PK/PD study of faricimab using PET/CT in an animal model of CNV.
• First report of faricimab intraocular distribution changes in healthy vs CNV eyes.
• Validation of CNV model as a translational tool for studying intravitreal antibodies.

## Graphical abstract

**Figure:**
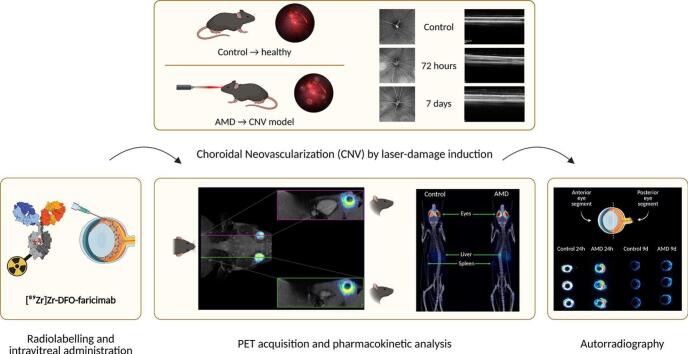

## 1 Introduction

Age-related Macular Degeneration (AMD) is a degenerative ocular disease affecting central vision and represents the leading cause of blindness in developed countries among people over 65 years (Campochiaro, 2015). Advanced AMD manifests as atrophic or neovascular AMD (nAMD), with nAMD being more severe and aggressive. At the molecular level, AMD develops from chronic oxidative stress, autophagy dysregulation, and chronic inflammation, leading to premature retinal pigment epithelium aging (García-Quintanilla et al., 2022). In turn, in nAMD, choroidal vascular dysfunction induces abnormal vessel growth, fluid accumulation, and activation of Vascular Endothelial Growth Factor (VEGF) (Aiello, 2008; Fragiotta et al., 2024).

Currently, intravitreal anti-VEGF therapy remains the standard treatment to inhibit VEGF activity and slow disease progression (Vemulakonda et al., 2025). These therapies improve anatomical and functional outcomes by suppressing neovascularization, reducing vascular leakage and exudation, and preserving visual function. Nevertheless, variability in treatment response, the burden of frequent injections, and associated adverse effects have prompted alternative therapeutic strategies. Faricimab, a bispecific humanized monoclonal antibody (mAb), represents a novel therapy for ophthalmic indications (Almuiña-Varela et al., 2024; Maguire et al., 2016; Panos et al., 2023; Singer et al., 2012). Designed to reduce immunogenicity and systemic exposure, faricimab simultaneously inhibits VEGF-A and angiopoietin-2 (ANG-2), offering broader control of neovascularization and vascular leakage than VEGF blockage alone (Agostini et al., 2024; Agrawal et al., 2025; Chujo et al., 2025; Heier et al., 2022).

Clinical Trial Real-world studies have shown that this dual mechanism translates into meaningful anatomical and functional improvements, sustained safety, and reduced treatment burden through extended dosing intervals, even in treatment-resistant patients (Heier et al., 2022; Wykoff et al., 2022). Available ocular pharmacokinetic data on anti-VEGF agents, including faricimab, are mainly inferred from aqueous humour or systemic concentrations, as direct vitreous sampling in humans is invasive and unfeasible (Diack et al., 2024a, Diack et al., 2024b). Consequently, most knowledge arises from preclinical studies. Traditional pharmacokinetic studies require sacrificing animals at multiple time points, introducing inter-subject variability and discontinuous data. In contrast, Positron Emission Tomography (PET) imaging enables real-time, non-invasive monitoring of drug distribution and longitudinal assessment of intravitreal pharmacokinetics, thereby reducing animal use in accordance with the 3Rs principles and offering an innovative alternative to conventional approaches (García-Otero et al., 2022; Luaces-Rodríguez et al., 2020).

The aim of this study is to characterise the pharmacokinetic profile of faricimab following intravitreal injection in a laser-induced Choroidal Neovascularization (CNV) rat model, using molecular imaging as a direct, non-invasive approach. In addition, retinal morphological changes are evaluated with Optical Coherence Tomography (OCT), and intravitreal antibody distribution is examined through autoradiographic imaging. To the best of our knowledge, this work represents the first study to report pharmacokinetic data of faricimab in an AMD model.

## 2 Materials and methods

### 2.1 Conjugation, biological activity, and radiolabelling

The radiolabelling of faricimab was performed following the protocol established by Verel et al. for other mAbs (Verel et al., 2003), which involves two distinct phases. In addition, quality control has been performed to verify that the antibody's functionality remained intact.

#### 2.1.1 Faricimab conjugation

Faricimab (Vabysmo®; Genentech, California, USA) was purified using 30 kDa Amicon® Ultra filters (Merck Millipore®, Massachusetts, USA). A two-fold molar excess of tetrafluorophenyl-N-succinyldeferoxamine-B-Fe^3+^ (DFO-Fe; ABX®, Radeberg, Germany) was added for conjugation, with the mixture incubated (25 °C, 50 min, pH 9.0–9.5). Following incubation, pH was reduced to 4.0–4.5 and a 50-fold molar excess of EDTA (ethylenediaminetetraacetic acid) was added to chelate the iron present in the DFO-Fe complex. It was then incubated again (35 °C, 45 min) and finally purified.

Conjugation quality was assessed by Size-Exclusion High-Performance Liquid Chromatography (SE-HPLC) on an Agilent 1260 series HPLC system (Agilent Technologies®, California, USA), enabling quantification of antibody aggregation and determination of the DFO/antibody ratio, following a previously established method (García-Otero et al., 2022).

#### 2.1.2 Biological activity of faricimab-DFO

An indirect ELISA was performed to evaluate the binding of faricimab-DFO to VEGF-A and ANG-2, assessing the preservation of its bioactivity and dual-target recognition capacity. 96-well half area clear flat bottom polystyrene high bind microplates (Corning 3690, Corning Inc. New York, USA) were coated overnight at 4 °C with 2 μg/mL VEGF-A (ab92846, Abcam, Cambridge, UK) and ANG-2 (10691-H08H, Sino Biological Inc., Beijing, China) peptides. After washing three times with PBST (PBS with 0.05% Tween20) and blocking for 1 h at 37 °C with 2% bovine serum albumin in PBS, various concentrations of faricimab and faricimab-DFO (8 to 0.0313 μg/mL) were added and incubated (2 h, 37 °C). The wells were washed three times with PBST and incubated at RT with goat-anti-human-IgG (Fc-specific) antibody horseradish peroxidase conjugate (A0170, Sigma Aldrich, Massachusetts, USA) for 1 h. After washing three times, K-Blue Aqueous TMB Substrate Solution (331175, Neogen, Michigan, USA) was added to the wells for colour development, incubated for 10 min and the reaction was stopped by adding 1 N HCl. The absorbance was measured at 450 nm using Victor Nivo Microplate Reader (Perkin Elmer, Massachusetts, USA).

#### 2.1.3 Faricimab radiolabelling

Radiolabelling was performed following the same protocol described in previous studies (García-Otero et al., 2022). Briefly, Faricimab-DFO was radiolabelled using clinical-grade [^89^Zr]Zr-oxalate (Revvity, Inc., Massachusetts, USA) by adjusting the pH to 4.0–4.5 with 2 M Na_2_CO_3_ and HEPES buffer to pH 7, and then incubated (RT, 1.5 h, specific activity: 10 MBq/mg). The radiolabelled antibody ([^89^Zr]Zr-DFO-faricimab) was concentrated and purified by ultrafiltration and radiochemical purity was quantified by trichloroacetic acid (TCA) precipitation, measuring radioactivity in protein-bound versus unbound fraction using a well counter (Atomlab Wipe Test Counter; Biodex, New York, USA).

### 2.2 Age-related Macular Degeneration (AMD) animal model

#### 2.2.1 Induction of Choroidal Neovascularization (CNV) Using laser in rats

**Fig. 1:**
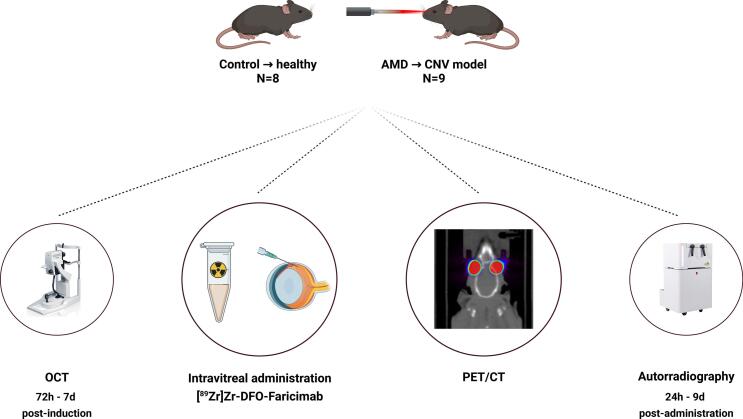
Experimental scheme of the different assays. Created with BioRender.com.

The study complied with the European Community guidelines for ethical use of laboratory animals (Directive 2010/63/UE) and approved by the University of Navarra Animal Research Committee (013–24) in accordance with ARRIVE guidelines. Seventeen males Brown-Norway rats (12 weeks-old, Charles River, Massachusetts, USA) were used and housed in standard cages under a 12-h light/dark cycle with food and water ad libitum. Anaesthesia was induced with xylazine (10 mg/kg; Xilagesic 2%; Calier Laboratories, Barcelona, Spain) and ketamine (75 mg/kg; Imalgene 1000; Merial Laboratories, Barcelona, Spain). Pupillary dilation was achieved with mydriatic eye drops. CNV lesions were induced in 9 rats (AMD group) following Garcia-Layana et al. protocol with modifications (García-Layana et al., 2009), (3–4 spots around the optic nerve, 532 nm, 250 mW, 0.05 s, 50 μm spot size). Rupture of Bruch's Membrane was confirmed by immediate bubble formation and vitreous haemorrhage, or absence of a bubble were excluded. Fig. 1 illustrated the experimental design of this work.

#### 2.2.2 Optical Coherence Tomography (OCT) characterisation

**Fig. 2:**
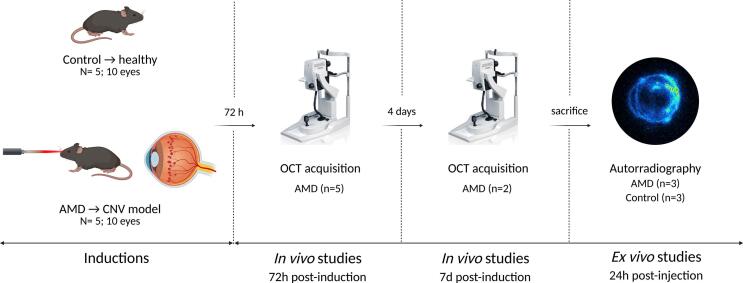
Experimental design of the OCT characterisation. Created with Biorender.com.

Ten Brown-Norway rats were used to evaluate retinal structural changes using OCT. Animals were divided into two groups: healthy control (Control, *n* = 5, 10 eyes) and AMD model (AMD, n = 5, 10 eyes) (Fig. 2). OCT assessments were conducted at the Animal Research Centre of the University of Santiago de Compostela (CEBEGA, Santiago de Compostela, Spain).

Retinal changes were assessed using a Spectral Domain-OCT (SPECTRALIS® OCT, Heidelberg Engineering, Germany), focusing on qualitative evaluation of abnormal areas. Protocols included infrared (IR) fundus imaging and structural tomographic scans. Assessments were performed 72 h post-induction and repeated 7 days post-induction in two AMD rats. For optimal image quality, eyes were dilated with mydriatic agent, fitted with a rigid gas-permeable contact lens. Animals were placed in prone positions on a heated platform on the OCT.

Volumetric scans were centred on the optic nerve (30° field, 121 B-scans, 49 μm spacing; 768 × 496 pixels per image over a 7.4 × 1.9 mm area). Eye-tracking software (TruTrack, Heidelberg Engineering, Germany) ensured consistency between time-points and intergroup comparisons were made using posterior segment images from Controls as reference.

### 2.3 PET pharmacokinetic and biodistribution studies

#### 2.3.1 Intravitreal administration of [^89^Zr]Zr-DFO-faricimab

Rats were anaesthetised with 3% (*v*/v) isoflurane (Baxter®, Illinois, USA) and maintained at 2.5% via face mask. Intravitreal injections were performed 72 h after model induction under a surgical microscope (Takagi OM-5220–2; Tokyo, Japan), following a previously described protocol (Fernández-Ferreiro et al., 2017). The 72-h timing aligns with peak vitreous VEGF expression according to Kim et al. (Kim et al., 2010) and Ben-Arzi et al. (Ben-Arzi et al., 2025). Topical anaesthetic drops and mydriatic drops were applied before injections. Each eye received 4 μL of [^89^Zr]Zr-DFO-faricimab (1–1.37 MBq or ± 0.18 mg of radiolabelled antibody), injected via pars plana with a 35 G needle using a NanoFil® syringe (WPI, Friedberg, Germany). Eyes with lens damage or intraocular bleeding during injection were excluded.

#### 2.3.2 PET/CT, PET/MRI and blood sampling

**Fig. 3:**
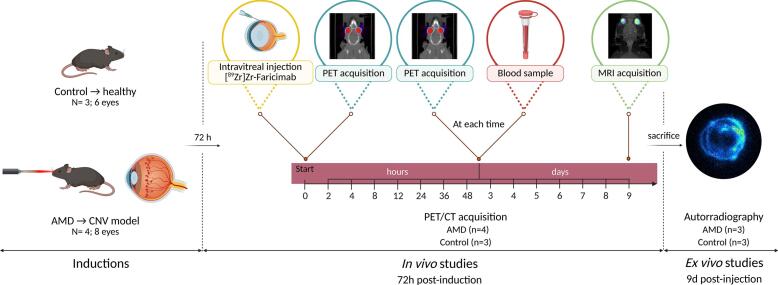
Study timeline for both groups (healthy and AMD group), intravitreal injections of [^89^Zr]Zr-DFO-faricimab, PET acquisitions, blood sample collection and autoradiography. Created with BioRender.com.

PET/CT scans were performed using an Albira PET/CT system (Bruker Biospin, Massachusetts, USA) immediately following intravitreal administration. Sequential two-bed scans (10 min each) were acquired under 2.5% isoflurane anaesthesia at multiple time points (0, 2, 4, 8, 12, 24, 36 h, then daily to day 10) to stablish the faricimab pharmacokinetic profile. PET data were reconstructed using the Maximum Likelihood Expectation Maximization algorithm (12 iterations, 0.5 × 0.5 × 0.5 mm^3^ image voxel). Corresponding Computerized Tomography (CT) images were acquired using four-bed positions (35 kV, 200 μA, 250 projections per bed), reconstructed at a resolution of 0.5 × 0.5 × 0.5 mm^3^. The combined PET/CT field of view covered the entire body of the animals. A schematic representation of the PET/CT study experimental design is provided in Fig. 3.

PET/MRI scans were performed at the end of PET/CT scans to provide complementary high resolution anatomical information and determine specifically the antibody's localization within the eye. PET/MRI static acquisition consisted of 10-min PET scan followed by a 11-min MRI scan using a Bruker BioSpec 3–T PET/MR scanner (bore diameter 17 cm), equipped with actively shielded gradients (450–900 mT/m). PET data was reconstructed using the MLEM algorithm (18 iterations, 0.5 mm voxel size) including scatter, randoms and decay correction. MRI was generated using a coronal head scan and performed using a Rapid Acquisition with Relaxation Enhancement (RARE) sequence (Echo Time = 12 ms, Repetition Time = 4.46 s, RARE Factor = 10, Averages = 8, 30 slices of 0.5 mm, 45 × 45 mm^2^ field of view and matrix size = 192 × 192).

Blood radioactive samples were collected before each PET and measured in the well counter. Total blood radioactivity was estimated using theoretical blood volume, calculated as proposed by Lee and Blaufox (Lee and Blaufox, 1985) and expressed as the percentage of [^89^Zr]Zr-DFO-faricimab uptake, normalised to the injected dose and body weight.

#### 2.3.3 Image analysis

PET images were analysed using Amide's software. Ocular uptake was quantified by drawing ellipsoidal Regions of Interest (ROIs) of 12 × 12 × 12 mm (904 mm^3^) encompassing the whole eye. The ROIs were intentionally defined as larger than the rat eye in order to compensate for partial-volume effects and the limited spatial resolution of small-animal PET imaging. The radiotracer concentration measured in the first image frame post-injection was used as reference. Subsequent measurements were expressed as a percentage of this initial uptake. Additional ROIs (7 × 7 × 7 mm, 343 mm^3^) were placed over the liver, spleen, and cervical lymph nodes to evaluate systemic distribution. All measures were decay-corrected based on the physical half-life of Zr-89 (3.3 days). Percentage of injected dose per organ (%ID/organ) was calculated by normalizing the mean [^89^Zr]Zr-DFO-faricimab activity in each ROI to the injected dose and organ volume.

#### 2.3.4 Pharmacokinetic analysis

The percentage of the remaining activity in the eye was fitted to the open two-compartment model described in our previous works (García-Otero et al., 2022, García-Otero et al., 2025; Varela-Fernández et al., 2025), whose equations model are (Eq. (1)):

(1) $$X_{1}=\frac{D\left(\beta -k_{12}\right)}{\beta -\alpha }\exp^{-\mathrm{\alpha t}}+\frac{D\left(k_{12}-\alpha \right)}{\alpha -\beta }\exp^{-\mathrm{\beta t}}$$

$$X_{2}=\frac{Dk_{12}}{\beta -\alpha }\left(\exp^{-\mathrm{\alpha t}}-\exp^{-\mathrm{\beta t}}\right)$$

where X_1_ represents the faricimab activity (expressed as % of remaining activity) in the eye compartment, D refers to the total administered activity, X_2_ represents the faricimab activity in the peripheric compartment, α and β are the hybrid constants of the model, and k_12_ is the micro-constant of distribution between the eye (compartment 1) and the blood and well-perfused organs (compartment 2). The hybrid constants can be expressed as (Eq. (2)):

(2) $$\alpha +\beta =k_{12}+k_{21}+k_{20}$$

$$\alpha \cdot \beta =k_{12}\cdot k_{21}$$

where k_21_ and k_20_ are the distribution micro-constant from the compartment 2 to the eye and the elimination constant from compartment 2, respectively.

The same model was used for the analysis of [^89^Zr]Zr-DFO-faricimab distribution in blood and organs, assuming that these belong to the peripheral compartment. Nonlinear fitting was performed with weighting schemes based on the predicted concentration. The suitability of the model was assessed by analysing observed versus predicted concentration–time curves, the Akaike Information Criterion (AIC), and the percentage coefficient of variation (CV%).

### 2.4 Autoradiography

A total of thirteen animals (7 AMD and 6 Control) were used for autoradiography studies. Prior to euthanasia by carbon dioxide inhalation, [^89^Zr]Zr-DFO-faricimab was administered, and tissues were collected either 24 h or nine days post-injection (Fig. 2, Fig. 3). Sagittal sections (20 μm thick) were obtained using a cryostat (Leica CM1520, Leica Biosystems Nussloch GmbH, Nussloch, Germany) and mounted on SuperFrost™ Plus Adhesion Slides Microscope (Epredia™, Breda, Netherlands). Autoradiography images were acquired over 10-h using a BeaQuant® real-time quantitative autoradiography system and analysed with Beamage® v.3.5.2 and Beavacq® v.2.1.6 software (Ai4r, Nantes, France).

## 3 Results and discussion

### 3.1 Conjugation, biological activity, and radiolabelling

Conjugation of DFO-Fe to faricimab was performed twice. HPLC analysis indicated an average of 1.42 ± 0.02 DFO chelating groups per mAb molecule. The efficiency of iron (Fe^3+^) removal from the DFO was 85.3% ± 2.4. No dimers formation was observed for DFO-faricimab (0%).

**Fig. 4:**
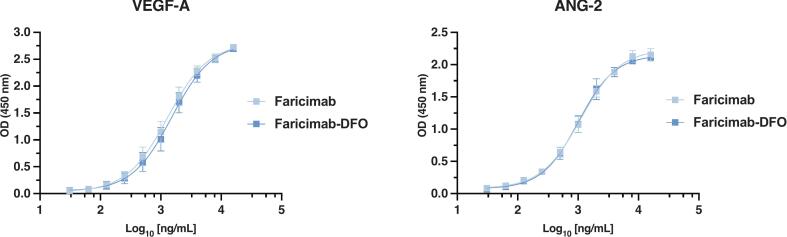
Bioactivity analysis of commercial faricimab compared with the faricimab-DFO conjugate against the two studied targets, VEGF-A and ANG-2.

To verify that the conjugated antibody maintains binding specificity to their targets, an indirect ELISA using VEGF-A and ANG-2 peptides was performed (Fig. 4). The results showed no significant differences in affinity between unconjugated faricimab and its DFO-conjugated form, with immunoreactivity values of 90.45% for VEGF-A and 99.2% for ANG-2, confirming that antibody functionality was preserved following conjugation.

Radiolabelling of DFO-faricimab with Zr-89 yielded a 93.55% radiochemical efficiency, which was further increased to 99.78% radiochemical purity after ultrafiltration.

### 3.2 Age-related Macular Degeneration (AMD) animal model

#### 3.2.1 Induction of Choroidal Neovascularization (CNV) and OCT characterisation

**Fig. 5:**
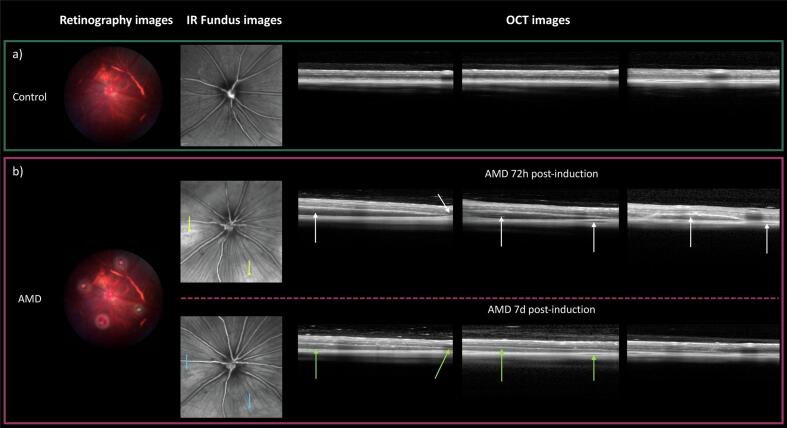
Retinography, IR fundus images and structural cross-sectional OCT scans of the different experimental groups, a) Control group, b) AMD 72 h and 7 days post-induction.

The correct induction of retinal lesions was confirmed by fundus imaging through retinography, showing well-defined white luminous foci around the optic nerve without haemorrhages immediately after induction (Fig. 5). After verifying the retinal lesions, the creation of CNV and retinal conformational changes were confirmed using OCT imaging.

As can be seen in Fig. 5b, the fundus image taken 72 h after induction shows the onset of an early CNV, characterised by hyperreflective areas corresponding to laser impacts (yellow arrows) which decreases 7 days after induction (blue arrows). In relation to OCT images, heterogeneous hyporeflective areas can be observed as localised subretinal oedema bordering the lesions 72 h after induction (white arrows). Finally, at 7 days post-induction, two signs can be identified: the reduction of these oedematous areas and retinal restructuring (green arrows), and the appearance of a turbid, hyperreflective vitreous chamber, suggestive of inflammation.

### 3.3 PET pharmacokinetic and biodistribution studies

#### 3.3.1 Ocular distribution and pharmacokinetics

Table 1 presents the pharmacokinetic fit parameters derived from applying two-compartment models to the ocular concentration data. The experimental data from both groups demonstrated a strong fit to the model, with comparable goodness-of-fit metrics. Table 1 also summarizes pharmacokinetic parameters associated with the elimination of intravitreal faricimab. The half-lives corresponding to the distribution (t₁/₂_α_) and elimination (t₁/₂_β_) phases were similar between the control group (4.77 and 48.48 h, respectively) and the AMD group (4.24 and 41.25 h), with no statistically significant differences. This indicates that [^89^Zr]Zr-DFO-faricimab resides in the ocular compartment for a comparable duration in both healthy and AMD-affected rats. Comparison of the area under the ocular activity–time curve (AUC_₀–∞_) revealed no significant differences between the control group (5587.28 ± 1419.32%·hour) and the AMD group (4079.08 ± 1178.58%·hour), although the values in the AMD group were slightly lower.

**Table 1:** Fitting results of intravitreally administered [^89^Zr]Zr-DFO-faricimab concentrations in the eye using a two-compartment model.

**Parameter**	**Control**		**AMD**	
	***Mean***	***SD***	***Mean***	***SD***
α	0.2714	0.2099	0.1837	0.1245
β	0.0147	0.0029	0.0191	0.0073
t_1/2α_ (hours)	4.77	4.57	4.24	2.06
t_1/2β_ (hours)	48.48	9.86	41.25	16.38
AUC_0-∞_ (%·hour)	5587.28	1419.32	4079.08	1178.58
MRT (hours)	68.86	7.10	61.46	10.71
R^2^	0.9986		0.9979	
S_y.x_	1.395	0.394	1.662	0.684
RMSE	1.236	0.349	1.473	0.606
AICc	21.036	8.327	25.338	11.033

α: distribution rate constant; β: elimination rate constant; AICc: Akaike Information Criterion; AUC_₀–∞_: area under the activity–time curve; MRT: Mean Residence Time; t₁/₂_α_: half-live corresponding to distribution phase; t₁/₂_β_: half-live corresponding to elimination phase; R^2^: correlation coefficient; RMSE: Root Mean Square Error; S_y.x_: standard error of estimate.

**Fig. 6:**
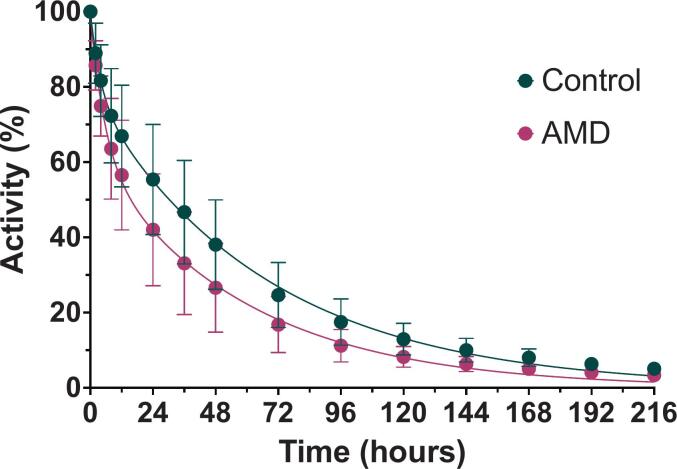
Percentage of remaining radioactivity in the eye versus time after intravitreal injection of [^89^Zr]Zr-DFO-faricimab in both rat groups (Healthy *n* = 6 eyes and AMD *n* = 8 eyes).

Quantitative analysis of the ocular PET images is also presented in Fig. 6, indicating no statistically significant differences in the kinetic curves between healthy and AMD rats. A progressive decline in ocular uptake is evident in both groups throughout the study. However, there is a noticeable trend suggesting faster clearance of the antibody in the AMD group.

As illustrated in Fig. 6, both healthy and diseased animals exhibited biphasic (two-compartment) pharmacokinetic behaviour, characterised by a brief initial distribution phase followed by a slower elimination phase.

**Fig. 7:**
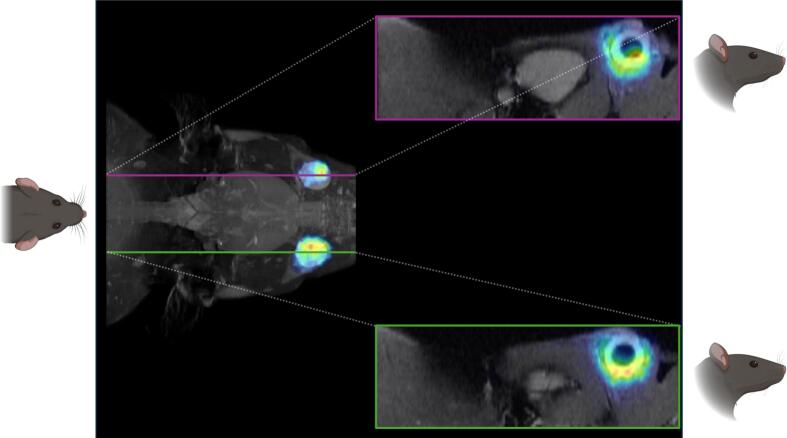
Fused PET/MRI image of the rat's head in coronal plane (left) and two sagittal plane images (right) after 9 days of distribution of [^89^Zr]Zr-DFO-faricimab injected intravitreally.

Fig. 7 illustrates the ocular distribution of intravitreally injected [^89^Zr]Zr-DFO-faricimab, as visualised by radioactive uptake 9 days after administration. The high-resolution images enable precise *in vivo* visualization of faricimab ocular distribution, showing its predominant localization within the vitreous humour and clearly delineating the anterior and posterior eye segments.

#### 3.3.2 Whole-body biodistribution

**Fig. 8:**
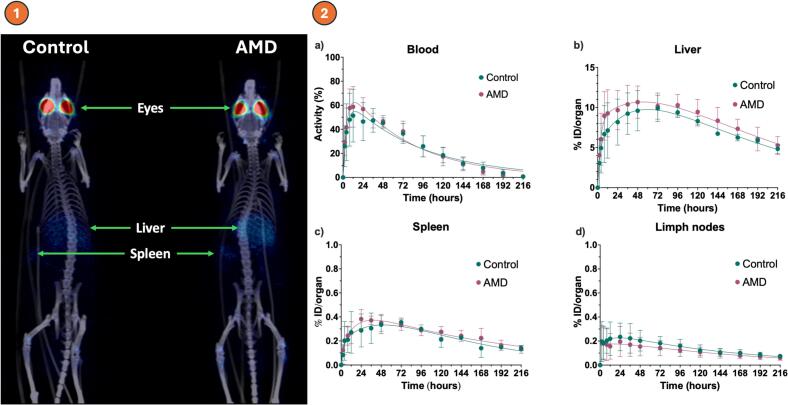
1) Coronal whole-body PET/CT images acquired 6 days after intravitreal injection of [^89^Zr]Zr-DFO-faricimab for both groups. The images illustrate the biodistribution of faricimab across different organs. Radioactive uptake is represented on a colour scale, with blue indicating lower intensity and red indicating higher intensity. 2a) Percentage of remaining radioactivity after intravitreal injection of [^89^Zr]Zr-DFO-faricimab in blood. 2b) Percentage of injected dose after intravitreal injection of [^89^Zr]Zr-DFO-faricimab in liver, 2c) spleen and 2d) lymph nodes versus time in both groups (Healthy *n* = 3 rats and AMD *n* = 4 rats). (For interpretation of the references to colour in this figure legend, the reader is referred to the web version of this article.)

Fig. 8(1) illustrate the whole-body distribution of [^89^Zr]Zr-DFO-faricimab 6 days post-intravitreal injection after its elimination from the eye into the systemic circulation. The highest faricimab uptake is observed in the liver and spleen. Fig. 8(2a) represents the blood uptake values (Activity (%)) of [^89^Zr]Zr-DFO-faricimab for both groups during the entire study period. A peak in blood activity occurs within the first 12 h (t_max_), followed by gradual distribution to tissues and organs involved in antibody clearance and catabolism, such as the liver and spleen (Fig. 8(b-c)), which mediate uptake, degradation, and immune regulation. There were no significant intergroup differences in kinetic curves or parameters (AUC_₀–∞_, t_max_) within the blood, liver, spleen, or lymph nodes.

### 3.4 Autoradiography

**Fig. 9:**
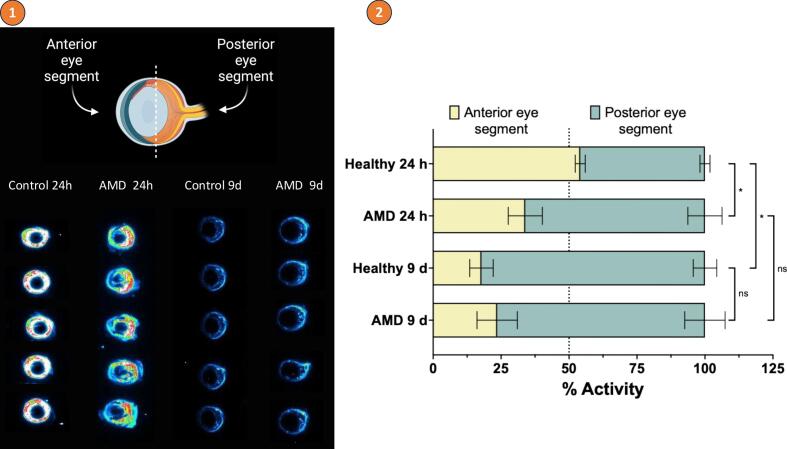
Representative image of the qualitative and semiquantitative autoradiographic analysis. 1) Histological slices acquired in the different groups analysed: control and AMD groups 24 h and 9 days post-administration of [^89^Zr]Zr-DFO-faricimab. 2) Semiquantitative analysis with the percentage of ocular activity in the anterior and posterior eye segments in the different groups.

Qualitative and semiquantitative analysis of autoradiographic images confirmed the effective intravitreal delivery and distribution of the radiolabelled antibody (Fig. 9), showing a notably higher uptake of [^89^Zr]Zr-DFO-faricimab in the posterior segment of the eye.

The semiquantitative analysis (Fig. 9(2)) revealed a significant difference in antibody uptake in the posterior segment between the control and AMD group at 24 h post-administration, with a higher proportion of activity in the AMD group. Interestingly, only the healthy control group showed a proportion of [^89^Zr]Zr-DFO-faricimab exceeding 50% in the anterior segment at 24 h while the AMD group displayed a markedly lower proportion, approximately 34%. After 9 days post-injection, although no differences were observed between two groups, the activity in the anterior segment had decreased relative to the posterior segment. At this stage, no differences were observed between the two groups, as most of the antibody had already been cleared from the eye, with the small remaining fraction localised primarily in the posterior segment.

## 4 Discussion

Access to clinical ocular pharmacokinetic data for antibody therapies in nAMD is limited, relying largely on preclinical studies due to challenges in intraocular sample collection. This study aimed to characterise faricimab's pharmacokinetic using non-invasive imaging in a CNV animal model, complemented by the evaluation of retinal morphological changes and antibody distribution via autoradiography.

Faricimab was conjugated with DFO and radiolabelled with Zr-89. According to established criteria by Verel et al. and Lindmo et al. (Lindmo et al., 1984; Verel et al., 2003), antibodies are considered sufficiently immunoreactive when radiolabelled immunoreactivity exceeds 70–80%. Our binding assays demonstrated that DFO-conjugation preserved faricimab's function, yielding strong affinity comparable to the native antibody, with immunoreactivity values of 90.45% for VEGF-A and 99.2% for ANG-2. A potential limitation inherent to ^89^Zr-DFO-based immuno-PET tracers is the partial *in vivo* dissociation of ^89^Zr^4+^ from the chelator, which may contribute to delayed uptake in the liver and spleen. This phenomenon has been previously described and generally attributed to transchelation of released ^89^Zr to plasma proteins or tissue components (Verel et al., 2003). While no dedicated *in vivo* stability assay was performed in the present study, our group has previously demonstrated at least 168 h of stability in human serum for [^89^Zr]Zr-DFO immunoconjugates generated using the same conjugation and radiolabelling procedure (Codesido et al., 2025), supporting the robustness of the methodology in this work.

Pathological neovessel formation in CNV is driven by factors such as oxidative stress and inflammation (Spaide et al., 2020). In this study, CNV model was successfully induced by laser disruption of Bruch's membrane, triggering the damage-induce response and subsequent CNV (Edelman and Castro, 2000; Hoerster et al., 2012; Khan et al., 2023; Liu et al., 2013; Meyer et al., 2019; Miller et al., 1990; Nakagawa et al., 2018; Salas et al., 2023). Fundus and OCT imaging revealed subretinal oedema consistent with fluid extravasation, previously correlated with fluorescein leakage by Albert D.M et al. (Albert et al., 2010). Although fluorescein angiography is the gold standard for confirming and characterising neovessels, our OCT findings supports the presence of pathological neovessels, mimicking clinical nAMD (Bhutto and Lutty, 2012; Campochiaro, 2015; Schmidt-Erfurth et al., 2014). As described by Salas A. et al., lesions peaked around day 7 followed by partial regression (Salas et al., 2023), while inflammatory signs resembling AMD. This inflammatory response, involving ANG-2 pathway activation (Agostini et al., 2024; Chen and Xu, 2015; Joussen et al., 2021), supports the model's suitability for pharmacokinetic evaluation of faricimab, a bispecific mAb targeting VEGF-A and ANG-2 (Heier et al., 2022).

Pharmacokinetic analysis revealed that faricimab elimination fits a two-compartment model, with no significant differences in ocular residence time between groups indicating that disease status does not substantially impact pharmacokinetics. Our findings align with Diack et al. (Diack et al., 2024b), who also reported no differences in ocular kinetics between healthy and retinal disease patients. Compared with our prior studies using bevacizumab and aflibercept (Luaces-Rodríguez et al., 2020), faricimab displayed longer ocular residence than aflibercept in healthy animals, while AUC was comparable to bevacizumab. Nevertheless, these comparisons should be interpreted cautiously, as faricimab was studied in pigmented Brown-Norway rats, whereas bevacizumab and aflibercept data came from albino Sprague-Dawley rats, which differ in ocular permeability (Gao et al., 2002; Zhang et al., 2005). To accurately compare these antibodies, it is necessary to conduct these studies using the same strain and within the same experimental model. Future studies should address this point to enable more robust and directly comparable pharmacokinetic assessments.

Monoclonal antibody ocular clearance occurs primarily by diffusion into the anterior chamber through the vitreous humour (del Amo et al., 2017). *Ex vivo* autoradiography at 24 h and 9d post-injection confirmed predominant activity in the posterior segment, particularly in CNV eyes at 24 h, suggesting delayed anterior diffusion across the vitreous humour. This pattern may be explained by target-mediated drug disposition, whereby binding may contribute to target antigens influences antibody distribution (Cao and Jusko, 2014). In the case of faricimab simultaneous binding to VEGF-A and ANG-2 and formation of antigen-antibody complexes may contribute to delayed intraocular redistribution. In contrast, healthy eyes showed faster anterior diffusion, indicating a greater contribution of aqueous humour elimination. By day 9, most antibody activity had cleared, with residual signal confined to the posterior segment, consistent with the distribution reported for other intravitreally administered monoclonal antibodies (García-Otero et al., 2022; Luaces-Rodríguez et al., 2020). High-resolution PET/MRI imaging further corroborated this posterior localization, providing a detailed and non-invasive visualization of faricimab distribution *in vivo*. PET/MRI studies could therefore be used in future ocular pharmacokinetic studies, as it allows for a more precise definition of the ocular compartments.

From a translational perspective, the present study may offer a valuable framework for future comparative evaluation of different antibodies and advanced drug delivery systems. By enabling the identification of strategies that enhance ocular bioavailability and prolong intraocular exposure, this approach may ultimately support the development of therapies capable of achieving extended dosing intervals in clinical practice.

## 5 Conclusions

This study provides the first *in vivo* characterisation of the intravitreal pharmacokinetics and intraocular distribution of faricimab in a rat model of laser-induced choroidal neovascularization. By employing autoradiography, valuable insights into the intraocular distribution of the antibody were obtained, thereby making a significant contribution to the field of ophthalmology.

PET/CT imaging demonstrated a biphasic ocular clearance profile in both healthy and AMD animals, with similar half-lives and residence times, suggesting that disease status does not significantly alter intravitreal faricimab pharmacokinetics. However, AMD eyes showed a trend toward faster systemic diffusion, likely reflecting BRB disruption and neovascularization. Autoradiography confirmed predominant retention in the posterior segment, with higher early accumulation in AMD eyes. Systemic biodistribution revealed expected uptake in the liver and spleen, comparable to other intravitreally administered antibodies.

Overall, this work validates PET-based molecular imaging as a real-time non-invasive and reliable tool for longitudinal assessment of ocular pharmacokinetics, offering new insights into intravitreal behaviour of faricimab in health and disease. These findings strengthen the rationale for its clinical use in AMD and open the possibility of evaluating and comparing novel therapeutic strategies aimed at increasing ocular residence time, while also highlighting the translational relevance of imaging-guided pharmacokinetic studies.

## CRediT authorship contribution statement

**Xurxo García-Otero:** Writing – review & editing, Writing – original draft, Visualization, Validation, Methodology, Investigation, Formal analysis, Data curation, Conceptualization. **Andrea Cuartero-Martínez:** Writing – review & editing, Writing – original draft, Visualization, Validation, Methodology, Investigation, Formal analysis, Data curation, Conceptualization. **Patricia Fernández-Robredo:** Writing – review & editing, Writing – original draft, Visualization, Validation, Methodology, Investigation, Conceptualization. **Soraya Groba-de Antas:** Writing – review & editing, Methodology, Investigation, Formal analysis, Data curation. **Noemí Gómez-Lado:** Writing – review & editing, Methodology. **Francisco J. Otero-Espinar:** Writing – review & editing, Visualization, Supervision, Formal analysis. **María Hernández:** Writing – review & editing. **Jaione Bezunartea:** Writing – review & editing. **Alfredo García-Layana:** Writing – review & editing, Validation, Resources. **Sergio Recalde:** Writing – review & editing, Visualization, Validation, Supervision, Resources, Funding acquisition, Conceptualization. **Pablo Aguiar:** Writing – review & editing, Visualization, Validation, Supervision, Resources, Funding acquisition, Conceptualization. **Anxo Fernández-Ferreiro:** Writing – review & editing, Visualization, Supervision, Resources, Project administration, Funding acquisition, Conceptualization.

## Funding sources

This work was partially supported by Ministry of Science and Innovation of Spain (MICINN) [PID2022-142350OB-C21] and [PID2025-169967OB-I00], Axencia Galega Innovación [ED431C 2025/08, IN607A2023/04, IN607D-2021/01]. Furthermore, this work was partially supported by grant PI23/00069 from the 10.13039/501100004587Instituto de Salud Carlos III (ISCIII) and co-funded by 10.13039/501100000780European Union (EU) and Inflammatory Diseases Network (RICORS-REI) [RD24/0007/0015]. X. García-Otero and S. Groba-de Antas acknowledges the support of 10.13039/501100010801Xunta de Galicia through the postdoctoral and predoctoral fellowships [ED481B-2023-063 and ED481A-2022/392], respectively. A. Cuartero-Martínez is grateful to the Health Research Institute of Santiago de Compostela (IDIS, Spain) for financing her postdoctoral research fellowship [IDISPOS1782/2024].

## Declaration of competing interest

The authors report no declarations of interest. The authors are only responsible for the content and writing of this article.

## Data Availability

Data will be made available on request.
